# Supplementary material for: Anomalous efficiency elevation of quantum-dot light-emitting diodes induced by operational degradation
Source: Nat Commun. 2023 Nov 27;14:7785. doi: 10.1038/s41467-023-43340-w (PMC10682488; doi:10.1038/s41467-023-43340-w)
Supplement: Supplementary file 1 — Supplementary Information [file 41467_2023_43340_MOESM1_ESM.pdf]

# Supplementary Information

# Anomalous efficiency elevation of quantum-dot light-emitting diodes induced by operational degradation

Siyu He[1], Xiaoqi Tang[1], Yunzhou Deng[2,3]*, Ni Yin[4], Wangxiao Jin[1], Xiuyuan Lu[1], Desui Chen[1], Chenyang Wang[1], Tulai Sun[5], Qi Chen[4]* and Yizheng Jin[1]*

[1]Key Laboratory of Excited-State Materials of Zhejiang Province, State Key Laboratory of Silicon Materials, Department of Chemistry, Zhejiang University, Hangzhou 310027, China.

[2]State Key Laboratory of Modern Optical Instrumentation, College of Optical Science and Engineering, International Research Center for Advanced Photonics, Zhejiang University, Hangzhou 310027, China.

[3]Cavendish Laboratory, University of Cambridge, CB3 0HE, Cambridge, UK.

[4]i-Lab, CAS Key Laboratory of Nanophotonic Materials and Devices, Suzhou Institute of Nano-Tech and Nano-Bionics, Chinese Academy of Sciences, Suzhou 215123, China.

[5]Center for Electron Microscopy, State Key Laboratory Breeding Base of Green Chemistry Synthesis Technology and College of Chemical Engineering, Zhejiang University of Technology, Hangzhou 310014, China.

*Corresponding author: Dr. Yunzhou Deng (yd359@cam.ac.uk), Prof. Qi Chen (qchen2011@sinano.ac.cn) or Prof. Yizheng Jin (yizhengjin@zju.edu.cn).

# Table of Contents

**1. Supplementary Figure**

Supplementary Figures 1-14

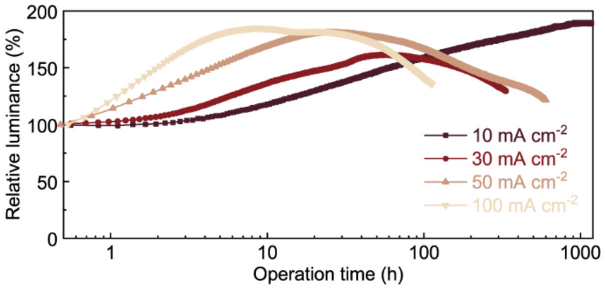


**Supplementary Fig. 1 | Time evolutions of the luminance of the shelf-stable QLEDs driven at different current densities.**

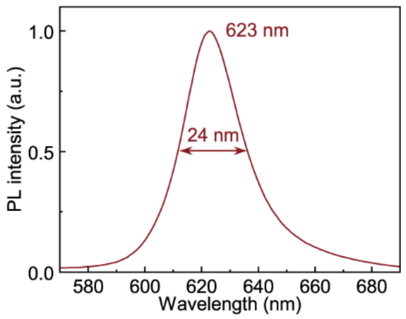


**Supplementary Fig. 2 | PL spectrum of the QD film.** The single-exciton (**X**) emission of the QD film features a peak wavelength of 623 nm and a FWHM of 24 nm.

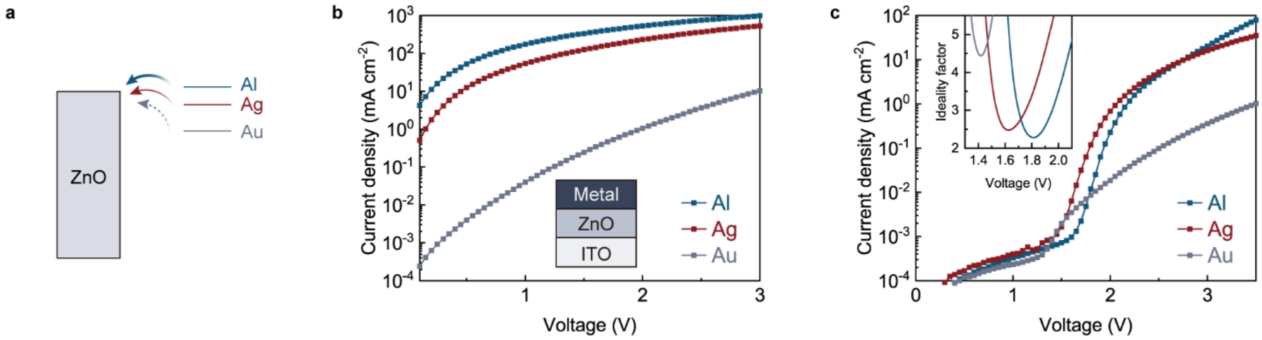


**Supplementary Fig. 3 | Impact of the electron-injection capability on the ideality factor. a,** Schematic diagram of electron injection from three different cathode metals to the ZnO ETL. Al electrode offers the lowest electron-injection barrier, and the Au electrode the highest electron-injection barrier. **b,** J-V curves of the electron-only devices with Al (blue), Ag (red) or Au (grey) cathodes. **c,** J-V curves of the QLEDs with Al (blue), Ag (red) or Au (grey) cathodes. Inset: ideality factors extracted from the J-V curves. The results demonstrate that poor electron injection leads to a larger ideality factor.

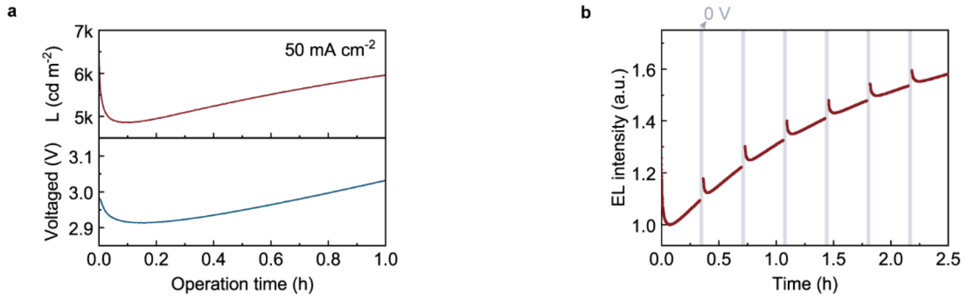


**Supplementary Fig. 4 | Temporal behaviours of the QLED at the initial stage of operations driven by a constant current. a,** Luminance (red curve, top) and driving voltage (blue curve, bottom) evolutions of the QLED at the initial stage of operation driven by a constant current of 50 mA $cm^{-2}$. **b,** EL intensity evolutions of the QLED under pulsed electrical stressing, alternating between 20 min of constant current (100 mA $cm^{-2}$) and 2 min of relaxation (grey-shaded regions, bias: 0 V). The rapid decay in EL intensity repeatedly occurs at the early stage of turn-on after every relaxation period. In contrast, the long-term efficiency-elevation process corresponds to irreversible changes in the steady-state of the QLED.

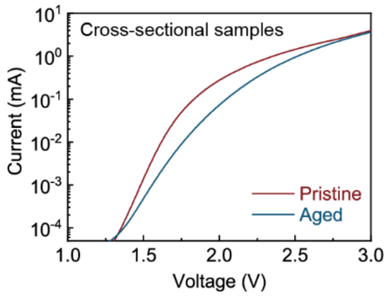


**Supplementary Fig. 5 | J-V measurements on the cross-sectional samples of the pristine (red curve) and the aged (blue curve) QLEDs.** The aged cross-sectional device shows a decreased slope in the J-V curve, confirming the preservation of the electrical properties of the pristine and the aged devices.

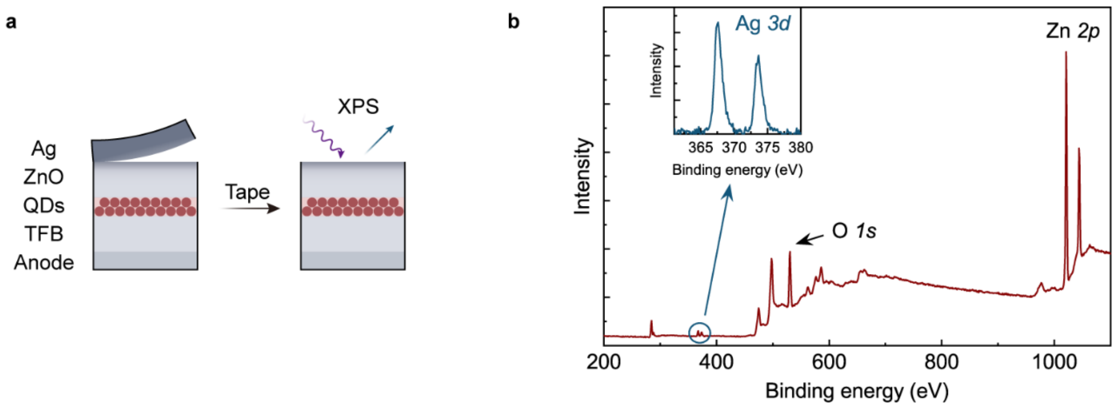


**Supplementary Fig. 6 | XPS characteristics of Ag-removed device. a,** Schematics of XPS investigations on the Ag-removed device. **b,** The survey scan and the high-resolution scan of the device. The weak Ag *3d* signal (blue circle and inset for high-resolution spectrum) indicates the residue of the interfacial species.

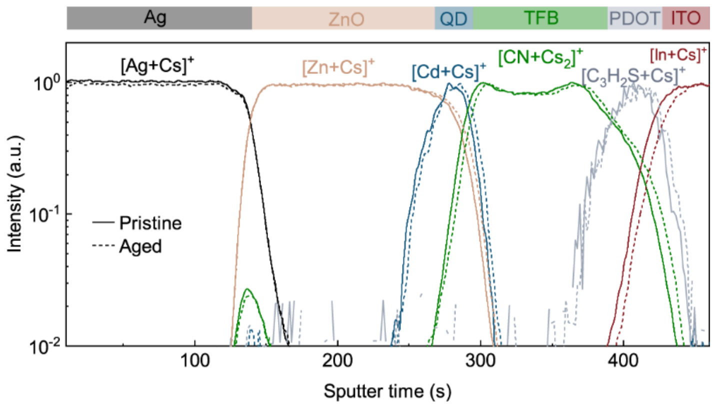


**Supplementary Fig. 7 | ToF-SIMS profiling of the QLEDs before and after efficiency elevation.** The results of a pristine QLED and an aged QLED are denoted by the solid curves and the dashed curves, respectively.

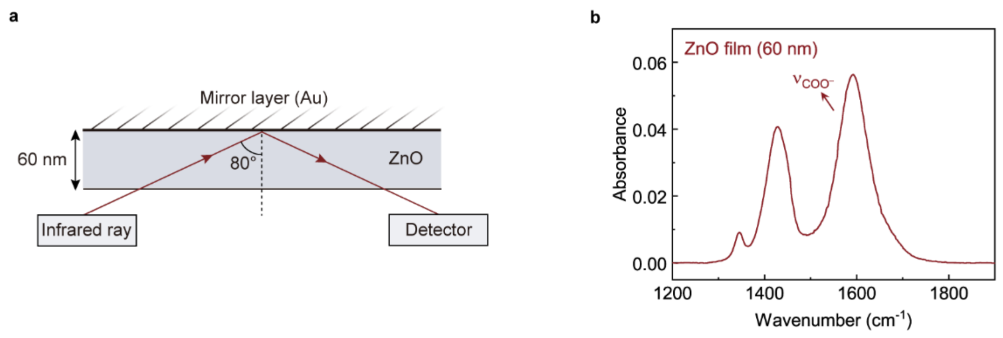


**Supplementary Fig. 8 | Quantitative FTIR analyses of the acetate concentration within the ZnO film. a,** Schematic diagram of the reflection mode. An infrared ray passes through a ZnO nanoparticle film (60 nm) at an incident angle of 80° and then enters the detector by reflection. The equivalent light path (*b*) across the ZnO film is approximated as $6.9 \times 10^{-5}$ cm. **b,** The absorption spectrum of the acetates. The absorbance (*A*) corresponding to the C=O stretching vibration (1600 $cm^{-1}$) in the ZnO film is determined to be 0.056. The concentration of acetate (*c*) is then determined to be ~$1.8 \times 10^{-3}$ mol $cm^{-3}$ by solving the Lambert-Beer equation ($A = \varepsilon bc$), in which the molar absorption coefficient of acetate ($\varepsilon$) is taken from a previous literature[1] ($4.5 \times 10^{2}$ $M^{-1}$ $cm^{-1}$).

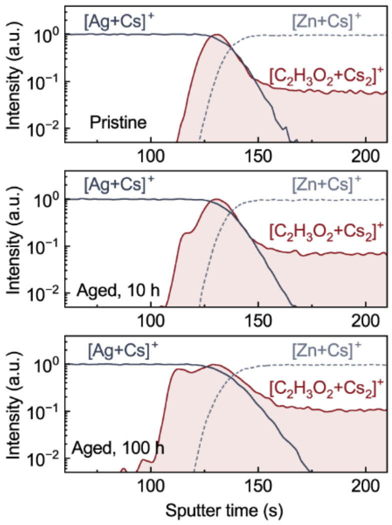


**Supplementary Fig. 9 | ToF-SIMS depth profiles of the ZnO/Ag interfaces in QLEDs aged to different stages.** The acetate shows a pronounced trend of migration towards Ag as the ageing time increases from 0 h to 100 h (stressed at a constant current density of 50 mA cm$^{-2}$).

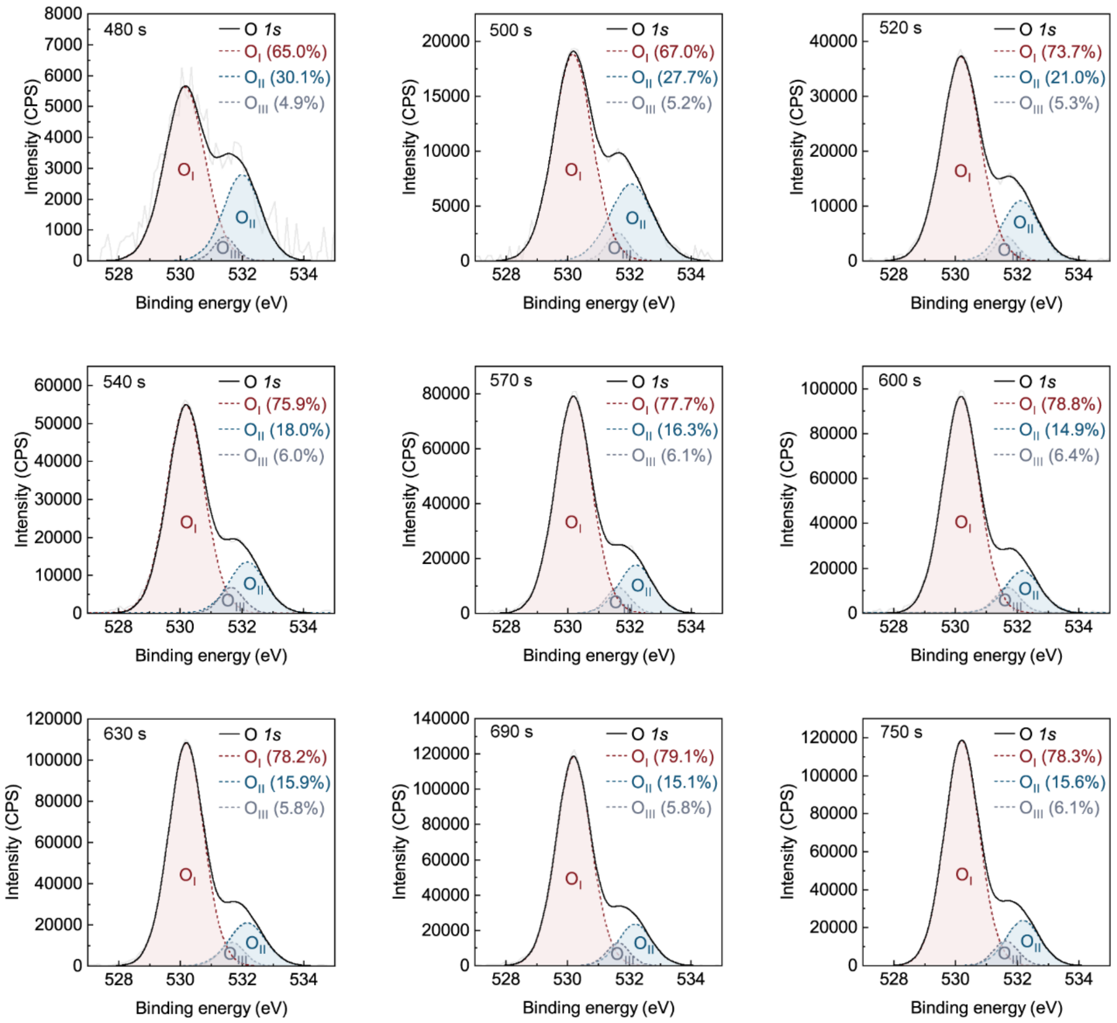


**Supplementary Fig. 10 | Gaussian fitting of the O *1s* spectra at different depths.** The corresponding etch times are labelled. A larger portion of the surface oxygen species (blue peak, $O_{II}$) is presented at the ZnO/Ag interface (short etch time).

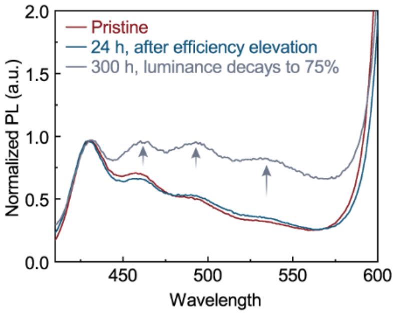


**Supplementary Fig. 11 | PL spectra of the TFB HTLs in QLEDs aged to different stages.** PL spectra show minimal changes (from red curve to blue curve) after the efficiency-elevation process (50 mA $cm^{-2}$ for 24 hours) but discernible enhancements in the lower-energy emissions (grey curve) after further ageing of this device to a stage with luminance drop to 75% of the initial value (50 mA $cm^{-2}$ for 300 hours).

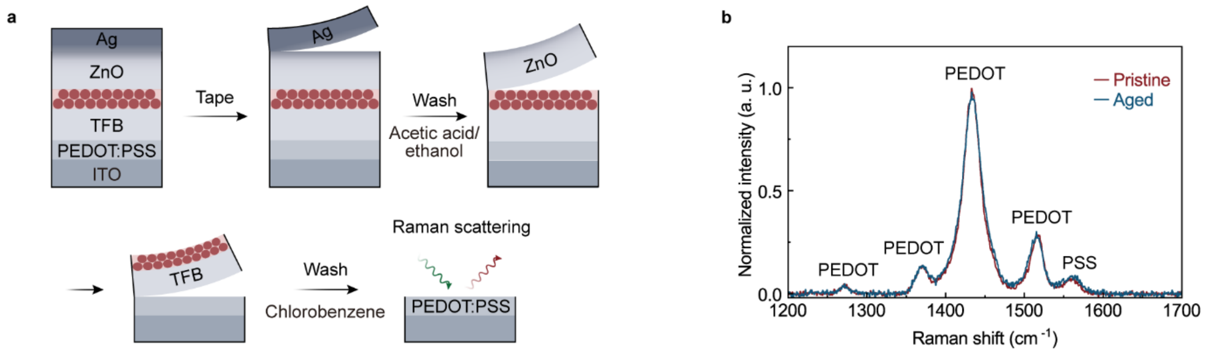


**Supplementary Fig. 12 | Raman spectra of the PEDOT:PSS HILs in the pristine and aged QLEDs. a,** Schematics of exposing the surfaces of PEDOT:PSS in the QLEDs by removing all the top layers for Raman characterizations (see Methods for details). **b,** Raman spectra of the PEDOT:PSS layers in the pristine (red curve) and aged (blue curve) devices.

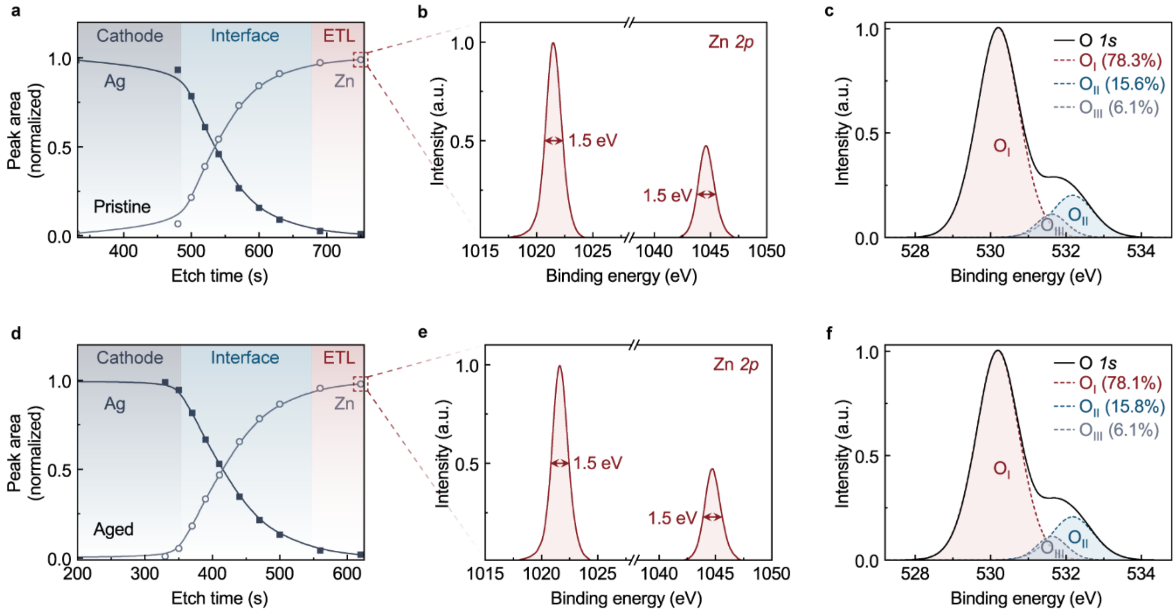


**Supplementary Fig. 13 | XPS analyses in the bulk of the ZnO ETLs in the pristine and aged QLEDs.** Depth profiles of the relative contents of Zn and Ag in pristine (**a**) and aged (**d**) QLEDs. The Zn *2p* spectra (**b**, **e**) and Gaussian fitted O *1s* spectra (**c**, **f**) are extracted from the bulk of the ZnO ETLs of pristine and aged devices, respectively. The Zn *2p* spectra show identical peak positions (Zn *2p3* at 1021.5 eV and Zn *2p5* at 1044.6 eV) and the similar FWHMs (full width at half maximum, 1.5 eV) before and after the efficiency-elevation process. The O *1s* spectra show similar compositions of the oxygen species with ~78% of $O_I$, ~16% of $O_{II}$, and ~6% of $O_{III}$ in these devices.

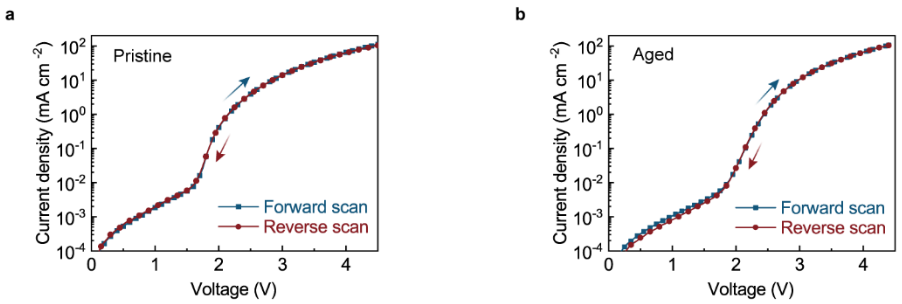


**Supplementary Fig. 14 | Forward and reverse J-V scans of the pristine and aged QLEDs.** J-V curves of the pristine device (**a**) and the aged device (**b**) obtained from forward scans (blue curves) and reverse scans (red curves). No resistance hysteresis is observed in the device.

## Supplementary Reference

1. Pike P. R., Sworan P. A. & Cabaniss S. E. Quantitative aqueous attenuated total reflectance Fourier transform infrared spectroscopy. Part II. Integrated molar absorptivities of alkyl carboxylates. *Anal. Chim. Acta* **280**, 253-261 (1993).
